# A mortality prediction model for critically ill patients with acute on chronic liver failure

**DOI:** 10.1186/2197-425X-3-S1-A973

**Published:** 2015-10-01

**Authors:** M Dalorzo, P Garcia Olivares, E Keough Delgado, C Bocanegra Abarca, I De Sousa, L Tang Rodriguez, N Fernandez Araujo, C Monis Cedano, JE Guerrero Sanz

**Affiliations:** H.G.U Gregorio Marañon, Intensive Care Unit, Madrid, Spain

## Introduction

Despite advances in critical care that have improved ICU outcomes in cirrhotic patients with acute decompensation they continue to present a high mortality.

## Objectives

Our aim was to obtain a mortality prediction model for patients admitted to the ICU with Acute on Chronic Liver Failure (ACLF), based on early clinical data.

## Methods

Retrospective study performed on patients admitted to our ICU with ACLF between 2010 and 2013. We collected epidemiological and clinical data in addition to scoring systems commonly used in critical care units and those more specific to cirrhotic patients.

Descriptive data were described as means with standard deviation for normally distributed continuous variables, medians with interquartile range (IQR) for non-normally distributed variables, and percentages for categorical data. The continuous variables were categorised according to maximum discrimination point by the area under the receiver operating characteristic (AUROC) curve.

The variables were compared using the Student t test for continuous variables, and the Chi-square test for categorical data. The predictive model was obtained by multiple logistic regression analysis.

## Results

Sixty-one patients, 79% male. Age 53 yrs (48 - 61). Charlson Comorbidity Index 4 pts (3 - 6). Severity scores: APACHE II 22 (18 - 25), SOFA 10 pts (8 - 13), MELD 19 pts (14 - 24), CLIF-SOFA 11 pts (9-1 4). 27% of patients had dysfunction of more than two organs. Alcohol abuse (55%) was the most frequent cirrhosis etiology and CHILD C (34%) the most prevalent cirrhosis stage. Reason for ICU admission: upper gastrointestinal haemorrhage 38%, hepatic encephalopathy 33% and sepsis 23%. 89% of patients required mechanical ventilation, 77% vasopressor therapy and 26% renal replacement therapy. Length of ICU stay 7 days (3-14) and 49.2% mortality in ICU.

In the **univariate analysis,** the variables related to mortality were: **CHILD C stage** (RR 4.64; CI 95% 1.47-14.63), **Admission diagnosis Sepsis** (RR 3.68; CI 95% 1.01-13.48), **dysfunction of more than two organs** (RR 8.37; CI 95% 2.06-34.03), **APACHE II > 22 pts** (RR 5.84; CI 95% 1.89-17.99), **MELD> 22 pts** (RR 7.56; CI 95% 2.16-26.45), **CLIF-SOFA> 12 pts** (RR 13.30; CI 95% 3.63-48.69), **vasopressor therapy** (RR 2.76; CI 95% 1.90-4.04) and **renal replacement therapy** (RR 2.14; CI 95% 1.96-3.14).

Using **multiple logistic regression,** we created a model which included the following variables: **Admission diagnosis sepsis** (OR 5.34; CI 95% 1.04-27.39), **dysfunction of more than two organs** (OR 4.50; CI 95% 0.75-26.79), **MELD> 22 pts** (OR 5.38; CI 95% 1.16-24.92), **APACHE II >22 pts** (OR 5.94; CI 95% 1.36-25.99).

**Figure 1:**
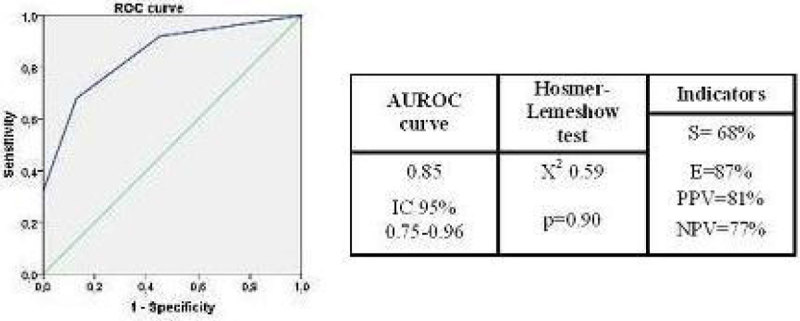
Figure 1

The model obtained had a good discriminatory capacity for patient prognosis: **AUROC 0.85; IC 95% 0.75-0.96** and **Hosmer-Lemeshow test; X**^**2**^**0.59, p = 0.90.** (Figure 1)

## Conclusions

In our experience, the predictive model created using clinical variables obtained early on ICU admission was able to discriminate the prognosis in patients with ACLF.

